# A Fast Algorithm to Estimate the Deepest Points of Lakes for Regional Lake Registration

**DOI:** 10.1371/journal.pone.0144700

**Published:** 2015-12-14

**Authors:** Zhanfeng Shen, Xinju Yu, Yongwei Sheng, Junli Li, Jiancheng Luo

**Affiliations:** 1 Institute of Remote Sensing and Digital Earth, Chinese Academy of Sciences, Beijing 100101, China; 2 Department of Geography, University of California, Los Angeles, CA 90095–1524, United States of America; 3 Xinjiang Institute of Ecology and Geography, Chinese Academy of Sciences, Urumqi 830020, China; NERC Centre for Ecology & Hydrology, UNITED KINGDOM

## Abstract

When conducting image registration in the U.S. state of Alaska, it is very difficult to locate satisfactory ground control points because ice, snow, and lakes cover much of the ground. However, GCPs can be located by seeking stable points from the extracted lake data. This paper defines a process to estimate the deepest points of lakes as the most stable ground control points for registration. We estimate the deepest point of a lake by computing the center point of the largest inner circle (LIC) of the polygon representing the lake. An LIC-seeking method based on Voronoi diagrams is proposed, and an algorithm based on medial axis simplification (MAS) is introduced. The proposed design also incorporates parallel data computing. A key issue of selecting a policy for partitioning vector data is carefully studied, the selected policy that equalize the algorithm complexity is proved the most optimized policy for vector parallel processing. Using several experimental applications, we conclude that the presented approach accurately estimates the deepest points in Alaskan lakes; furthermore, we gain perfect efficiency using MAS and a policy of algorithm complexity equalization.

## Introduction

Precise registration of images and lakes is required for lake change detection and analysis in the North American state of Alaska. To perform this task, many ground control points (GCPs), or tie points [1], are required. Because Alaska contains many lakes and is covered with ice and snow much of the year, it is very difficult to locate valid GCPs in multi-phase remotely sensed images or in lake extraction results. Despite the existence of many sophisticated registration algorithms, it is still difficult to register images acquired over such areas, owing to a dearth of stable features [1]. The shapes and areas of lakes change significantly over time, thus, it is necessary to locate the most stable points in the lakes as the GCPs. Sheng and Chintan proposed methods that use the centroids of stable lakes as tie points for automated image registration [1, 2]. Compared with centroids of lakes, we contend that the lakes’ deepest points are more suitable GCPs for image registration, because as lakes shrink or expand, the deepest point of the lake will remain the same until the lake dries up [3]. A lake can be seen as a specific shape of polygon; the boundary of the lake corresponds to the edge of a polygon, which can be used to compute the deepest point of a lake for use as a valid GCP [4]. The deepest point of a lake can be calculated by the distance from all the lakeshores to this inner point. Furthermore, from a mathematical point of view, the center point of its largest inscribed circle is the deepest point. Therefore, the problem of the deepest point estimation of a lake is indeed to find the circle center point of the largest inner circle (LIC) for an arbitrary polygon.

Many existing references focus on seeking LICs for *convex* polygons [5] or specific polygons [6], rather than arbitrary polygons. Reference [7] provides an iterative approach for locating the LICs of arbitrary polygons; however, there exist two main issues in the algorithm. First, a local maximum circle, rather than the global LIC, may be obtained because of different initial iterative points; second, the algorithm’s efficiency may be different when different iterative steps are selected, and several uncertain problems may also appear. The Voronoi diagram is an important mathematical method in computational geometry [8], and can describe the main skeleton of a polygon. It has a strict mathematical definition and calculation method, and the center point of the LIC must be an intersection of these Voronoi diagram lines or parabolas [9, 10]. Therefore, we can locate the LIC by calculating the Voronoi diagram of a polygon corresponding to a lake.

Furthermore, the medial axis (MA) of a polygon is also developed for the LIC problem, and its algorithm may be more efficient. The concept of medial axis transformation was first proposed by Blum in 1967 [11] as a means to describe a figure. It is defined as follows: given a polygon represented by *G*, the medial axis *M*(*G*) is the point set {*q*} internal to *G* such that there are at least two points on the object’s boundary that are equidistant from {*q*} and are closest to {*q*}. Hence, it is also referred to as a skeleton [11]. According to the definition above, an intersection of medial axes is equidistant to at least three edges or vertexes (both belong to *site* or *elements*); thus, the LIC’s center point must be one of the points from the medial axis intersection sets {*o*}. This technique has been proven useful in many engineering fields such as element analysis, form analysis, path planning in robotics, solid modeling, and mesh generation [12]. In the literature, different methods have been proposed for computing the medial axis either approximately or exactly, including relying on discrete geometry [13–16], digital topology [17,18], mathematical morphology [19], computational geometry [20,21], partial differential equations [22], or level-sets [23]. Generally, the methods for initial MA generation can be divided into three types, namely, thinning-based methods, tracing methods, and methods based on the Voronoi graph [24]. In this study, we obtain the medial axis result of a polygon by simplifying a Voronoi diagram [5–7].

To expand upon the lakes registration problem and its application in Alaska, the medial axis generation algorithm is first defined for an arbitrary vector polygon; based on this, the LIC seeking algorithm is also presented. In addition, to better manage the large number of lakes and large volumes of data, two improved methods for this algorithm are then respectively proposed—medial axis simplification (MAS) and parallel computing; both methods can improve the efficiency of the algorithm. The key issues involving the vector data partition policy were then discussed in detail; the algorithm complexity equalizer policy (ACEP), defined later in the paper, proved to be the most effective method for data partition. Finally, LIC-seeking experiments were conducted in Alaska; in these experiments, the algorithm proposed in this paper is proved to be feasible and efficient.

## Methods

### A largest inner circle-seeking algorithm for a vector polygon

The Voronoi diagram, which was defined in 1908 by Russian mathematician MG Voronoi, is a very important mathematical model in computational geometry; it has been widely applied in geometry, geography, and other fields [25–27]. We can obtain the polygon’s medial axis by simplifying the Voronoi diagram.

#### 1. Voronoi diagram generation algorithm based on “divide-and-conquer.”

**Fig 1:**
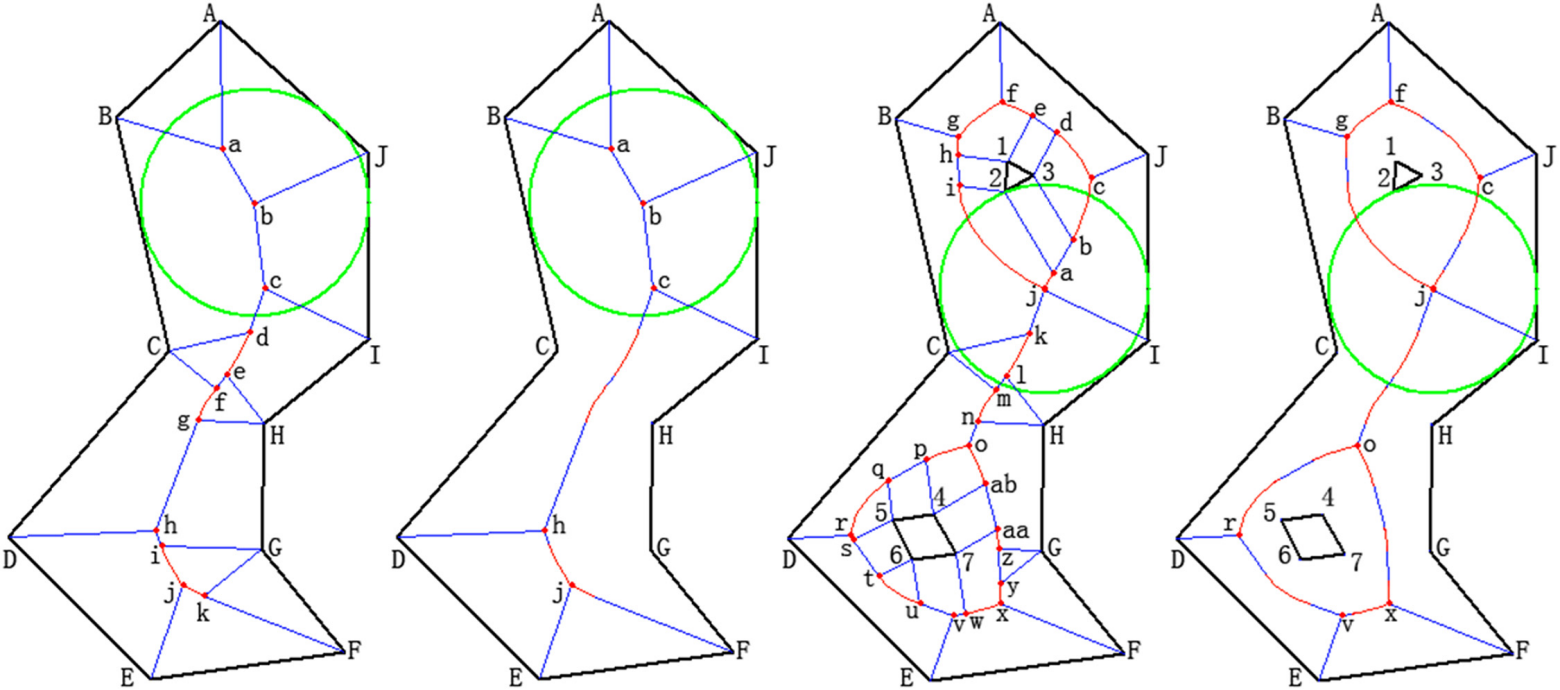
The algorithm to find the LIC based on Voronoi and medial axis. **(a)** Voronoi diagram of a simple polygon. **(b)** Medial axis diagram of a simple polygon. **(c)** Voronoi diagram of a complex polygon. **(d)** Medial axis diagram of a complex polygon.

The existing references use several classic Voronoi diagram generation algorithms; their algorithm complexities are respectively *o*(*n* ^2^) [28], $o(n\;c^{\sqrt{log\;n}})$ [29], *O*(*n*log^3^ *n*) [30], *O*(*n*log^2^ *n*) [10], *O*(log *m* + *n*log *n*) [31], *O*(*n*log *n*) [9,10,32] and so on, where *n* is the edge number of the polygon and *m* is the number of interior intersections. However, these references have not considered the existence and influence of interior islands for a polygon, and this is a very important and inevitable issue when studying changes in lakes. In this paper, an algorithm whose complexity is also *O*(*n*log *n*) is presented, and the influence of islands on algorithm complexity is analyzed in detail. We separate arbitrary polygons into two classes: simple polygons and complex polygons. Simple polygons have no interior island inside; in other words, the plane is divided into only two regions, interior and exterior. In contrast, complex polygons contain one or more islands inside. Fig 1 shows the algorithm implementation effect that locates the largest inner circle of a polygon.

In Fig 1, the black lines marked from A to J show the boundary of a lake; Fig 1(a) and 1(b) represent simple polygons, and Fig 1(c) and 1(d) represent complex polygons. The numbers from 1 to 7 in 1(c) and 1(d) denote nodes that represent islands. The lines in the polygon are the segments or parabolas generated by our algorithm (described in the following sections); blue lines represent segments and red lines represent parabolas. The points marked *a*,*b*,… are the intersections of the generated lines. Moreover, the green circle shows the largest inner circle sought by the algorithm described. We will introduce the principle and implementation procedure of the algorithm in the following sections.

**Fig 2:**
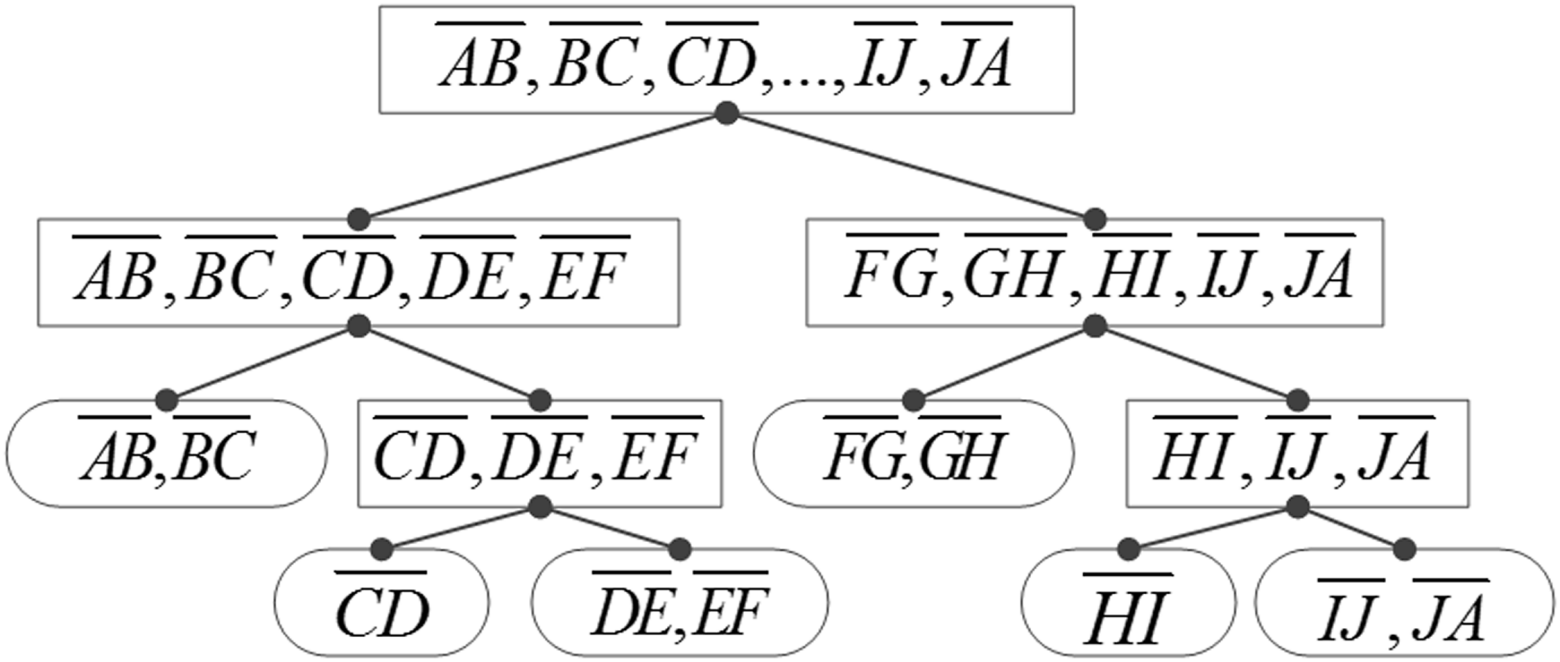
Voronoi generation tree based on “divide-and-conquer” method.

1.1 Voronoi diagram generation method for a simple polygon: Because there will be numerous generated Voronoi lines inside, and different algorithms may exhibit different levels of efficiency when generating a Voronoi diagram, we adopt the “divide-and-conquer” method to complete the Voronoi diagram. In Fig 1(a), the polygon consists of 10 edges: $\overset{-}{AB}$, $\overset{-}{BC}$, …, and $\overset{-}{JA}$. These are evenly divided into two groups, and each group is computed to complete the Voronoi diagram. Subsequently, the two results are merged and the final Voronoi diagram result is obtained. Moreover, the five edges in each group can also be divided using the method shown in Fig 2, until each leaf node has only one or two edges remaining.

As an example, consider the merging process of layer 2 in Fig 2. According to Figs 1(a) and 2, the Voronoi results should be merged from the second level to the first level of Fig 2; that is, the method used to merge segment $\overset{︹}{AB,BC,\ldots ,EF}$ and segment $\overset{︹}{FG,GH,\ldots ,JA}$. The following section (1.2) of this paper will discuss the algorithm. Using this method, we can obtain all the leaf node shows in Fig 2, and the next section (1.3) of this paper will describe the Voronoi generation method for the leaf nodes.

**Fig 3:**
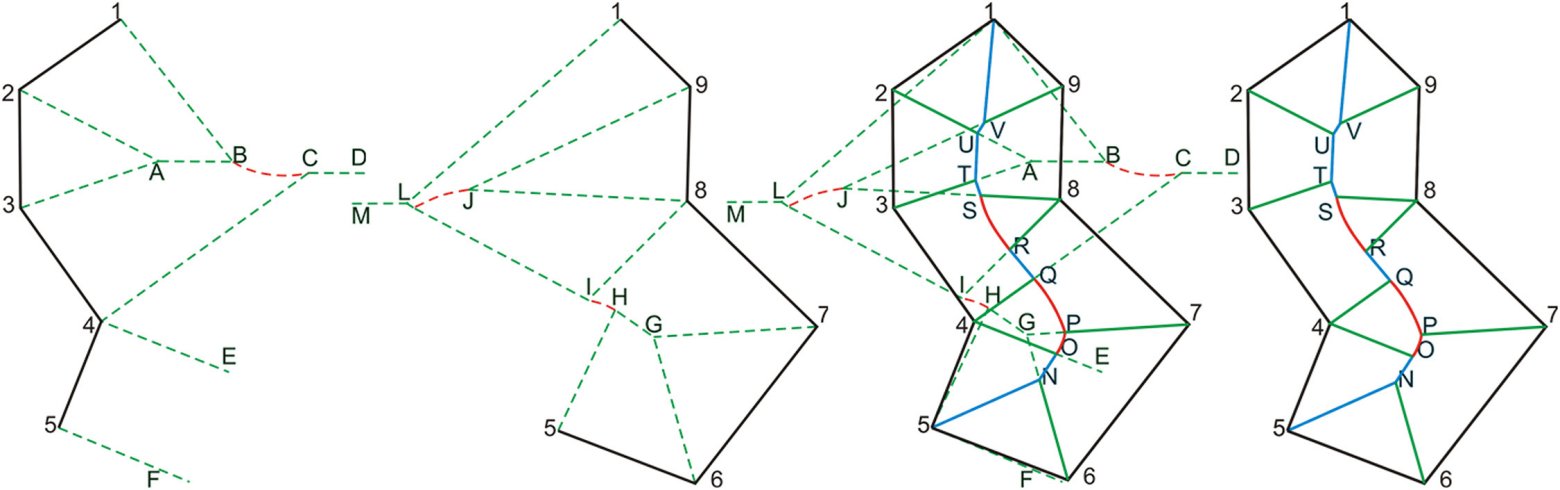
Voronoi diagram generation algorithm based on the binary tree principle for a simple polygon.

1.2 Method to merge the Voronoi diagram for simple polygons: For simple polygons, Voronoi diagrams can be calculated using the method shown in Fig 3, whose principle is similar to the divide-and-conquer procedure of a binary tree. According to Fig 2, a simple polygon can be divided into several Voronoi generation steps, and each step can be completed by the method described in this section and the following section. In Fig 3, the black lines represent edges or segments of the lake boundary, the green dashed lines are the generated Voronoi straight lines, rays, or segments, the red dashed lines correspond to the Voronoi parabolas, and the blue lines and red parabolas (in Fig 3(c) and 3(d)) are the merged final results generated by the proposed algorithm.

Fig 3 shows the Voronoi diagram calculation process of a simple polygon, which is also described in Fig 2. For a simple polygon with *n* edges, the Voronoi calculation process can be implemented with the following steps: First, the edges in the polygon are divided averagely into two parts (see Fig 3(a) and 3(b)), as shown in the first level of Fig 2. Second, each part’s Voronoi result is calculated separately and merged, as shown in Fig 3(c). The final Voronoi result shown in Fig 3(d) is then achieved. Similarly, the parts shown in Fig 3(a) and 3(b) can also be divided into two parts until each part only contains one or two sides, as shown in the last level of Fig 2, similar to a binary tree bifurcation procedure.

Fig 3(a) and 3(b) can be merged to achieve the result shown in Fig 3(c). The calculation procedure should start from an intersection of the two parts, e.g., point 1 or point 5. In this example, we start from point 5, and the angle of point 5’s two sides (∠456) should then be calculated. If the angle is acute, the bisector of the angle is the result (explained later in 1.3); if the angle is obtuse, the two perpendicular lines should be adopted. In this case, the bisector $\overset{-}{5N}$ of ∠456 is calculated; the sources of the two sides are also recorded, denoted here as $\overset{-}{5N}\leftarrow (\overset{-}{54},\overset{-}{56})$ (see Fig 3(c)). In the next phase, the other intersection of $\vec{5N}$ is judged after moving downwards. After calculating the intersection of segment $\vec{5N}$ with the Voronoi line from point 4 ($\overset{-}{4E}$) and point 6 ($\overset{-}{6G}$), the first intersection *N* is selected ($\overset{-}{5N}$ intersects with $\overset{-}{6G}$ at point *N* before intersecting with $\overset{-}{4E}$). As the process continues to judge the intersection sequence and record the corresponding sources, we obtain $\overset{-}{NO}\leftarrow (\overset{-}{54},\overset{-}{67})$. By searching in the same manner, $\overset{⌢}{OP}\leftarrow \left(4,\overset{↔}{67}\right)$ can be obtained, where $\overset{⌢}{OP}$ is a parabola generated from focus 4 and directrix $\overset{↔}{67}$. The above process continues until the other intersection of the two parts is found, e.g., point 1. Here $\overset{⌢}{PQ}\leftarrow \left(4,\overset{↔}{78}\right)$, $\overset{-}{QR}\leftarrow (\overset{-}{43},\overset{-}{78})$, $\overset{⌢}{RS}\leftarrow \left(8,\overset{↔}{43}\right)$, $\overset{-}{ST}\leftarrow (\overset{-}{43},\overset{-}{89})$, $\overset{-}{TU}\leftarrow (\overset{-}{32},\overset{-}{89})$, $\overset{-}{UV}\leftarrow (\overset{-}{21},\overset{-}{89})$, $\overset{-}{V1}\leftarrow (\overset{-}{21},\overset{-}{91})$ are calculated sequentially and the search procedure is completed.

According to Figs 2, 3(a) and 3(b) can be divided and calculated in the same manner, and the results are combined gradually as described in the procedure above. All the procedures are similar to the division and combination processes of the binary tree, and we refer to this procedure as a Voronoi generation process based on the "divide-and-conquer" technique. Section 1.3 shows some basic Voronoi diagram results generated by the leaf nodes of Fig 2.

**Fig 4:**
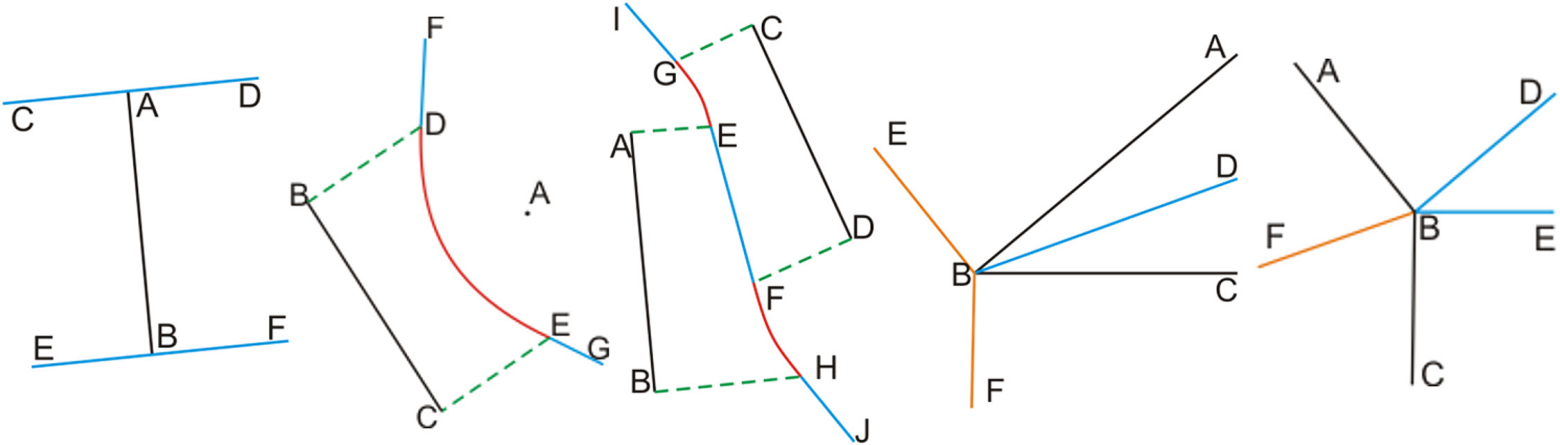
Several basic Voronoi diagram generation results. **(a)** Voronoi calculation method for a line segment. **(b)** Voronoi calculation method for a point and a line segment. **(c)** Voronoi calculation method for two segments. **(d)** Voronoi calculation method (∠*ABC* is an acute angle). **(e)** Voronoi calculation method (∠*ABC* is an obtuse angle).

1.3 Several basic Voronoi diagram generation methods: For the leaf of the “divide-and-conquer” binary tree, several basic Voronoi diagram generation methods are defined in Fig 4.

In Fig 4, black lines and points denote the input conditions (*sites* or *elements*), and blue and red lines are the generated Voronoi output results. Furthermore, blue lines represent straight lines, rays or segments, and red lines represent parabolas. Orange solid lines in Fig 4(d) and 4(e) represent the generated external Voronoi results, and the green dashed lines shown in Fig 4(b) and 4(c) are the lines perpendicular to the black input lines. Fig 4(a) is the Voronoi result of a line segment $\overset{-}{AB}$, and the results are two straight lines perpendicular to segment $\overset{-}{AB}$, denoted in this paper as $\overset{↔}{CD}\leftarrow (\overset{-}{AB},A)$ and $\overset{↔}{EF}\leftarrow (\overset{-}{AB},B)$ Fig 4(b) is the Voronoi result of a point A with a line segment $\overset{-}{BC}$ According to the definition of the Voronoi diagram, it is composed by the rays $\vec{DF}$ and $\vec{EG}$, and a parabola $\overset{⌢}{DE}$, where $\vec{DF}$ and $\vec{EG}$ are respectively the perpendicular bisectors of line segment $\overset{-}{AB}$ and $\overset{-}{AC}$, denoted in this paper as $\vec{DF}\leftarrow (A,B)$ and $\vec{EG}\leftarrow (A,C)$. Point *A* is the focus of parabola, and the straight line $\overset{↔}{BC}$ is the directrix of parabola, denoted here as $\overset{⌢}{DE}\leftarrow \left(A,\bar{BC}\right)$. Fig 4(c) is the Voronoi result of two segments $\overset{-}{AB}$ and $\overset{-}{CD}$, which consists of rays $\vec{GI}$ and $\vec{HJ}$, line segment $\overset{-}{EF}$, and two parabolas $\overset{⌢}{GE}$ and $\overset{⌢}{FH}$, where $\vec{GI}\leftarrow (A,C)$, $\vec{HJ}\leftarrow (B,D)$. Line segment $\overset{-}{EF}$ is the angle bisector of angle $\overset{-}{AB}$ and $\overset{-}{CD}$, which is expressed as $\overset{-}{EF}\leftarrow (\overset{-}{AB},\overset{-}{CD})$. Similarly, the other two parabolas can be expressed as $\overset{⌢}{GE}\leftarrow \left(A,\bar{CD}\right)$ and $\overset{⌢}{FH}\leftarrow \left(D,\bar{AB}\right)$. Fig 4(d) and 4(e) show the Voronoi results of two situations in which ∠*ABC* is respectively an acute angle and an obtuse angle. In Fig 4(d), because ∠*ABC* is an acute angle and point *B* is *convex*, its internal Voronoi result is the angle bisector $\vec{BD}$, denoted as $\vec{BD}\leftarrow (\overset{-}{AB},\overset{-}{BC})$, and its external Voronoi results are two perpendicular segments, expressed here as $\vec{BE}\leftarrow (\overset{-}{AB},B)$ and $\vec{BF}\leftarrow (\overset{-}{BC},B)$. In Fig 4(e), ∠*ABC* is an obtuse angle and point *B* is a *reflex* point, and its result is contrary to the case shown in Fig 4(d). For those simple polygons, the Voronoi diagram results refer only to the internal Voronoi lines (blue lines in Fig 4(d) or 4(e)). For the complex polygons, the internal Voronoi results of the outer ring and the external Voronoi results of the inner ring should be calculated and then combined into the final Voronoi results (see section 1.4).

1.4 Voronoi diagram for a complex polygon with Islands inside: For the complex polygons with internal islands, we should calculate not only the internal Voronoi diagram of the outer ring ($\overset{︹}{AB...JA}$) (see Fig 1(c)), but also the external Voronoi diagram of the inner ring ($\overset{︹}{123}$ and $\overset{︹}{4567}$). Moreover, we must calculate the Voronoi diagram result between the outer and the inner ring, and here we use Fig 1(c) as an example to explain the procedure of the algorithm.

We must select a point from the inner ring as the starting point (see Fig 1(c)); in this example, we use point 1: First, we find the edge in the outer ring of the polygon having the shortest distance to point 1, i.e., edge $\overset{-}{BC}$. Then, we can obtain the Voronoi diagram from point 1 and edge $\overset{-}{BC}$ according to Fig 1(c), and the result is a parabola $\overset{⌢}{ghi}$ (the arc $\overset{⌢}{hi}$ is substituted by segment $\overset{-}{hi}$ because of the subsequent searching process). As we continue to search downward in the same direction, we obtain the parabola $\overset{⌢}{gf}$; both sides are also recorded and denoted here as $\overset{⌢}{gf}\leftarrow (1,\overset{-}{AB})$. As this searching method is repeated, we obtain $\overset{⌢}{fe}\leftarrow (1,\overset{-}{AJ})$ (here, the intersection *f* of arc $\overset{⌢}{gf}$ and arc $\overset{⌢}{fe}$ should be computed), $\overset{-}{ed}\leftarrow (\overset{-}{13},\overset{-}{AJ})$, $\overset{⌢}{dc}\leftarrow (3,\overset{-}{AJ})$, $\overset{⌢}{cb}\leftarrow (3,\overset{-}{JI})$, $\overset{-}{ba}\leftarrow (\overset{-}{32},\overset{-}{JI})$, $\overset{⌢}{aj}\leftarrow (2,\overset{-}{IH})$ and so on. We then search in the reverse direction from point 1, and we obtain $\overset{-}{hi}\leftarrow (\overset{-}{12},\overset{-}{BC})$, $\overset{⌢}{ij}\leftarrow (2,\overset{-}{BC})$ and so on. At last, we combine the results and obtain the Voronoi diagram between the inner and outer rings, i.e., $\overset{︹}{abc...ja}$. When the outer ring has more than one inner ring, the algorithm above should judge the distance not only to the outer ring, but also to the other rings, because the existence of other inner rings might lead to a change in the Voronoi diagram results.

#### 2. Medial axis generation algorithm for a polygon

As mentioned above, the medial axis of the polygon can be obtained directly from its Voronoi diagram [5]. According to the definition of the medial axis [30], the MA can be obtained by removing the *reflex* vertices from the outer ring and *convex* vertices from the inner rings of its Voronoi diagram, Fig 1(b) and 1(d) are the respective medial axes of Fig 1(a) and 1(c). Because we have removed the two perpendicular lines from both the *reflex* vertices of the outer ring and the *convex* vertices of the inner rings, the intersection number of MA will be 2(*n*_*er*_ + *n*_*ic*_) less than that of the Voronoi diagram, where *n*_*er*_ is the number of *reflex* vertices on the outer ring and *n*_*ic*_ is the number of *convex* vertices on the inner rings. By removing some intersections that do not require computation, the efficiency of the center point seeking algorithm will be significantly improved (see section 3).

#### 3. Methods to find the largest inner circle

According to the definition and characteristics of the Voronoi diagram, the LIC center point of the polygon must fall on the intersection of the Voronoi diagram. In other words, we can seek the center point from the point *a*,*b*,*c*,…,*k* for Fig 1(a) or seek from point *a*,*b*,…,*ac* for Fig 1(c) (red solid points in Fig 1); the seeking method involves computing the minimum distance from these points to all the sides. For Fig 1(a), the distance from point *a* to the line segment $\overset{-}{AB}$ is equal to the distance from this point to $\overset{-}{BC}$ and $\overset{-}{JA}$. Therefore, the circle whose center is point *a* will be tangent to line segments $\overset{-}{AB}$, $\overset{-}{BC}$ and $\overset{-}{JA}$ (in some cases, the circle may touch one of their endpoints but will not intersect with them; for example, in Fig 1(a), the circle whose center is point *e* will be tangent to the segments $\overset{-}{HI}$, and intersect with point *C*). After seeking, we record the maximum distance as the radius of the LIC, and the point corresponds to the center point of LIC, namely, the deepest point in the lake. The pseudo code for the seeking steps is as follows (see Table 1):

**Table 1:** Algorithm to seek the LIC.

1	double maxRadius = 0;
2	for point(i) in all intersections
3	double minDist = 99999999
4	for segment(j) in all linesegments
5	compute dist(point(i), segment(j))
6	if(dist < maxRadius)
7	break and turn to next point(i)
8	if(dist < minDist)
9	minDist = dist
10	if(minDist > maxRadius)
11	maxRadius = minDist

The main idea of the above pseudo-code is to locate the shortest distance from all the intersections of the Voronoi diagram to all its edges. If the shortest distance is smaller than variable *maxRadius*, the algorithm should break and move to the next point (line 7 in Table 1); if it is larger than variable *maxRadius*, the coordinates of the center point should be updated with those of point (j). Using this technique, the center point of the LIC and its radius can be achieved.

A medial axis has homologous characteristics, in the same manner as a Voronoi diagram; thus, we can obtain the LIC by calculating the shortest distance from the interior intersection point sets, namely, points *a*,*b*,*c*,*h*,*j* in Fig 1(b) or points *c*,*f*,*g*,…,*x* in Fig 1(d), to all the edges. The maximum value in these shortest distances is the radius of the LIC. Compared with the seeking procedure of the Voronoi diagram, the medial axis has fewer intersections of the possible center points, which reduces seek iterations and improves the searching efficiency of the algorithm.

#### 4. Algorithm complexity analysis

The LIC-seeking process shows that the medial axis and the Voronoi diagram generation process have the same algorithm complexity. Here, we analyze the complexity of a Voronoi algorithm for a simple polygon with *n* edges, which are divided into multiple leaf nodes for computation, as shown in Fig 2. The level number is log_2_ *n*, and during every iteration, the algorithm must judge both sides of the node *n* times. Therefore, the algorithm complexity is 2*n* × log_2_ *n*, i.e., *O*(*n*log *n*).

Assume a complex polygon has *m* inner rings, and there are *n*_1_,*n*_2_,…,*n*_*m*_ edges for every inner ring and $n-\sum_{i=1}^{m}n_{i}$ edges on the outer ring. Consequently, we can compute the algorithm complexity and obtain the result $O((n-\sum_{i=1}^{m}n_{i})log(n-\sum_{i=1}^{m}n_{i}))$, *O*(*n*_1_ log *n*_1_), …, *O*(*n*_*m*_ log *n*_*m*_) for the outer ring, the first inner ring, …, and the *m th* inner ring. For the case of *m* = 1, when we compute the Voronoi diagram between the outer ring and inner ring, we must find the shortest distance between the points on the inner ring and outer ring only once, and the algorithm complexity is *n*−*n*_1_. The algorithm then searches in two directions until the Voronoi diagram is close, and the algorithm complexity of this procedure is 2×(*n*−*n*_1_). Therefore, the final algorithm complexity is *n*−*n*_1_ + 2×(*n*−*n*_1_), i.e. *O*(*n*). For the case of *m*>1, the difference is that, during the first iteration, the seeking algorithm should consider the relationship of different inner rings. Thus, the algorithm complexity is *n*−*n*_*cur*_ + 2×(*n*−*n*_*cur*_), and *O*(*n*), where *cur* is the current inner ring and *cur* ∈ (1,2,…,*m*). According to the analysis mentioned above, we can determine that the final algorithm complexity is $O((n-\sum_{i=1}^{m}n_{i})log(n-\sum_{i=1}^{m}n_{i}))+O(n_{1}\;log\;n_{1})+\ldots +O(n_{m}\;log\;n_{m})+O(n)+\ldots +O(n)$, which is also *O*(*n*log *n*). Therefore, the algorithm complexity of medial axis seeking is also *O*(*n*log *n*).

We now analyze the algorithm complexity of the LIC center point seeking procedure. According to the LIC seeking algorithm shown in section 3, if there are altogether *s* internal intersections in the Voronoi diagram, the final algorithm complexity will be *n* · *s*/2, because the complexity of the final algorithm has been reduced by approximately half, the loop will break and move to next loop (see line 7 of Table 1). For a simple polygon, the number of internal Voronoi intersections is at most *S*_*voronoi*_ = *n*_*convex*_ *−* 2 + 2(*n* − *n*_*convex*_) = 2*n* − *n*_*convex*_ −2, where *n*_*convex*_ is the number of *convex* vertices (acute angle seen from the interior), and the number of internal MA intersections is *S*_*medial axis*_ = *n*_*convex*_ − 2. For complex polygons with internal islands, the number of internal Voronoi intersections is at most $s_{voronoi}=n_{convex}-2+2(n-n_{convex})+2\times \sum_{i=1}^{m}n_{i}+n=3n+2\sum_{i=1}^{m}n_{i}-n_{convex}-2$, and the number of internal MA intersections is at most $s_{medial\;axis}=n_{convex}-2+2\times \sum_{i=1}^{m}n_{i}$. Therefore, the Voronoi diagram algorithm complexity is at most $O(n\cdot s/2)=O(\frac{3}{2}n^{2}+n\sum_{i=1}^{m}n_{i}-\frac{n_{convex}}{2}-1)$, i.e., *O*(*n* ^2^); and the algorithm complexity of MA is at most $O(n\cdot s/2)=O(\frac{1}{2}n\cdot n_{convex}-1+n\cdot \sum_{i=1}^{m}n_{i})$, also *O*(*n* ^2^).

### Two improved methods for the largest inner circle-seeking algorithm

#### 1. Medial axis simplification of a polygon

According to the algorithm complexity analysis above, for a given lake polygon, there are two methods that can improve the efficiency of algorithms in the deepest point seeking process. The first method is to reduce the number of polygon edges *n*, which will directly reduce the algorithm complexity of the entire process. However, this may also reduce the accuracy of lake polygons and their LICs. In some applications, we can simplify the polygon to a certain extent to achieve high efficiency under the premise of calculating accuracy. Another method to improve efficiency is to reduce the number of medial axis intersections. Thus, we present a medial axis simplification (MAS) method to solve this problem.

**Fig 5:**
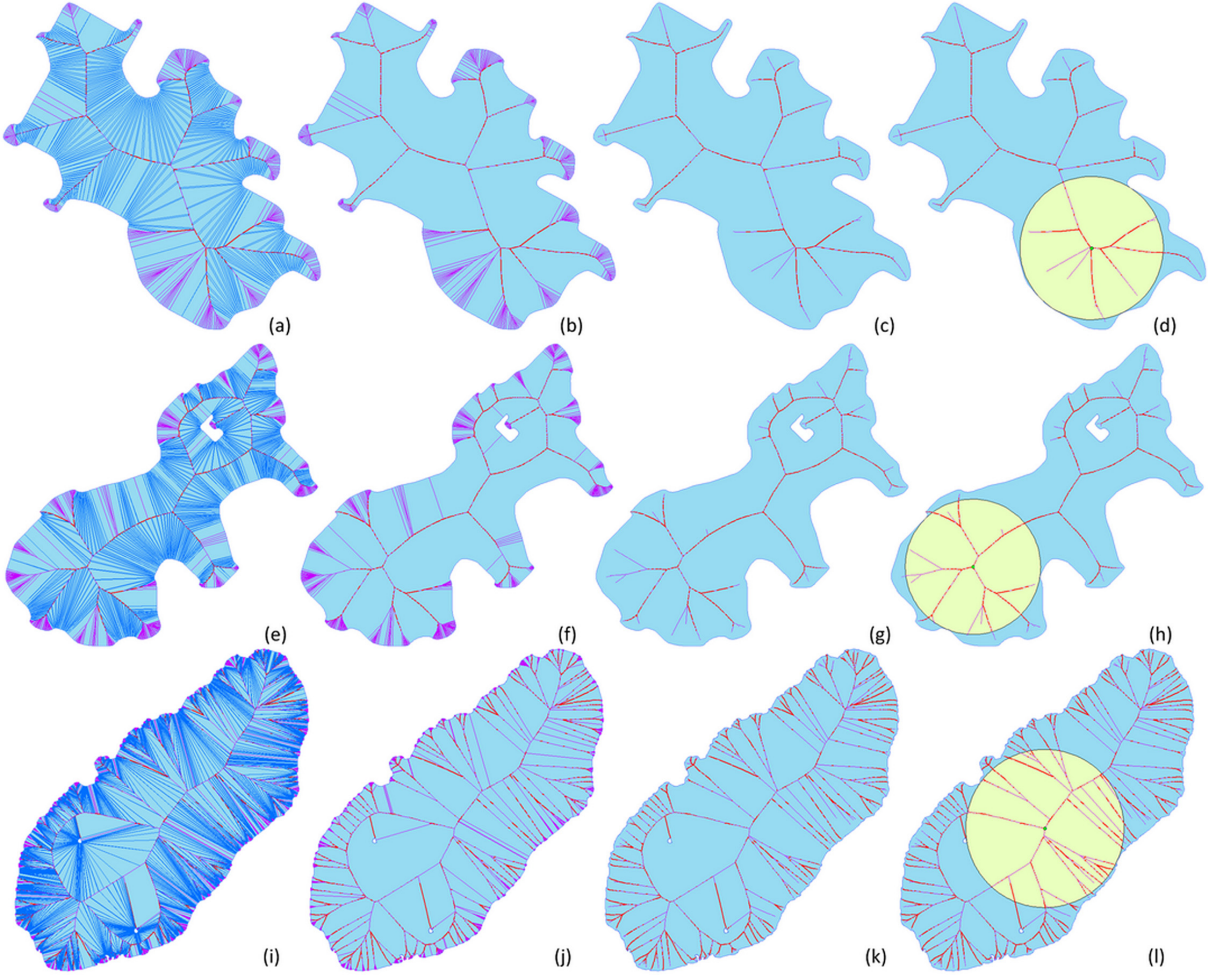
Comparison charts of medial axis simplification and LIC seeking.

In contrast with the Voronoi diagram method, the main idea of the MAS method is to reduce the number of intersections, which allows the LIC-seeking algorithm to reduce the scope of the searching process and increase seeking efficiency. In general, a lake is formed by many edges (i.e., it has a very large *n*), and this is mainly due to the vectorization process that uses remotely sensed images to extract lakes. Because the MA endpoints intersected with edges (*sites*) are very unlikely to represent the LIC center point for lake polygons [32], we can remove those medial axes that intersect with the edges; this can significantly improve algorithm efficiency. Fig 5 shows the results of the MAS method.

The red lines in Fig 5 are the generated Voronoi parabolas, the blue lines are the Voronoi segments passing through the *reflex* vertices on the outer ring or the *convex* vertices on the inner rings, and the purple lines represent the Voronoi diagram segments passing through the *convex* vertices on the outer ring or the *reflex* vertices on the inner rings. The first column of (Fig 5(a), 5(e) and 5(i)) shows the generated Voronoi diagram results of different lakes; the second column (Fig 5(b), 5(f) and 5(j)) shows the medial axis result; the third column (Fig 5(c), 5(g) and 5(k)) shows the results of MAS, and the last column (Fig 5(d), 5(h) and 5(l)) shows the LIC-seeking results based on the MAS shown in the third column. Table 2 lists the different intersection numbers in Fig 5. The results show that the MAS of a polygon can significantly reduce the possible center points to seek. Thus, efficiency can be improved by the MAS procedure.

**Table 2:** Intersection number comparison for MAS.

Intersection number	Voronoi intersections	MA intersections	MAS intersections
Fig 5(a), 5(b) and 5(c)	2343	1693	26
Fig 5(e), 5(f) and 5(g)	3588	2584	37
Fig 5(i), 5(j) and 5(k)	16072	11728	181

#### 2. Parallel computing and data partition policy

In addition to the above method of improving algorithm efficiency, we also adopt parallel computing to improve LIC-seeking algorithm efficiency. Currently, almost all computers have multi-core processors; ordinary algorithms cannot use multicore computing resources effectively. In this study, we transform the algorithm to effectively utilize multi-core computing. Because the LIC-seeking computing procedures for lake polygons are independent of each other, we may divide the features of vector data into several parts, and each part is calculated and completed in a computing core. Different data partition methods may lead to different acceleration ratios for the algorithm [33, 34]; thus, in this study we placed particular emphasis on the data partition policy for vector data.

The data partition policy of vector data is different from that of raster data; as such, it is important to establish an effective method to partition different features of vector data, to achieve balanced time consumption among different computing cores. For vector data, features are the smallest units to be processed, thus we can divide all the features (polygons) of the lake data into several parts to be computed. We propose three data partition policies: a sequential distribution policy, an attribute descending policy, and an algorithm complexity equalization policy. By comparing the acceleration ratios of different policies, we conclude that the algorithm complexity equalization policy (ACEP) is the optimal solution for parallel data partitioning and vector data calculations.

2.1 Sequential Distribution Policy (SDP): If there are *N*_*core*_ cores on the data processing computer and *N*_*feature*_ features (polygons) to be processed in the vector data, the data partition method of vector data should distribute *N*_*feature*_ features to *N*_*core*_ partitions; each vector data partition will be processed in a computing core. Here, we assume that there are a large number of features (polygons) to be processed (otherwise, parallel computing would not be necessary). Moreover, the Feature ID (FID) attribute of the vector data has no relationship with its edge number *n*. From the FID viewpoint, the edge number *n* is randomly distributed (its processing time is also randomly distributed). Therefore, we can use a simple feature partition method, called the sequential distribution policy (SDP), to divide all the features approximately evenly. Suppose the FID of all features is represented by $F_{1},F_{2},\ldots ,F_{N_{feature}}$, and the core numbers of the computer are 1,2,…,*N*_*core*_. We can distribute the features as follows: *F*_1_ ⇒1, *F*_2_ ⇒2, …, $F_{N_{core}}\Rightarrow N_{core}$, $F_{N_{core}+1}\Rightarrow 1$, $F_{N_{core}+2}\Rightarrow 2$, $F_{2\times N_{core}}\Rightarrow N_{core}$, …, until distribution is completed; the average feature number of every core is |NfeatureNcore|.

2.2 Attribute Descending Policy (ADP): In this section, we analyze the implementation procedure of the attribute descending policy (ADP). Although there are almost equal numbers of features in different cores, it is inevitable that some cores’ calculations are more complex than others (this is because calculations for features with a large number of edges *n* are more time-consuming). We solve this problem by applying an attribute descending method to the features. Attributes such as the area or perimeter of a feature can be easily obtained or computed (in this paper we use the OGR library function, OGRPolygon::get_Area()), and we use the area decreasing method on the assumption that computation time will decrease with a decrease in the area attribute. ADP works as follows: first, all the features will be sorted in descending order by their area attributes, and then these features are distributed to different cores back and forth in order to keep them as balanced as possible. After sorting in descending order, the FIDs of different features denoted as $F_{1},F_{2},...,F_{N_{feature}}$ satisfy $A_{F_{1}}\geq A_{F_{2}}\geq ...\geq A_{F_{N_{feature}}}$, where $A_{F_{i}}(i\in 1,2,...,N_{feature})$ is the area of feature *F*_*i*_. The feature distributing procedure is as follows: *F*_1_⇒1, *F*_2_⇒2, …, $F_{N_{core}}\Rightarrow N_{core}$, $F_{N_{core}+1}\Rightarrow N_{core}$, $F_{N_{core}+2}\Rightarrow N_{core}-1$, …, $F_{2\times N_{core}}\Rightarrow 1$,…, until the distribution is complete; the average number of features in a core is also |NfeatureNcore|.

2.3 Algorithm Complexity Equalization Policy (ACEP): Furthermore, we can improve the data partition policy on the basis of SDP and ADP. If we sort the features not according to their area or perimeter, but according to a new attribute *cpx*, which denotes the algorithm complexity described above, the policy’s feasibility increases because the computation demands on different cores are more balanced. ACEP works as follows: first, we compute a new attribute, algorithm complexity, with the equation *cpx* = *n*log *n* +n^2^/2, where *n*log *n* is the algorithm complexity of medial axis generation and n^2^/2 is the LIC-seeking complexity. We then sort the polygons by the new attribute *cpx* in descending order, and partition these features according to the complexity sum (*sum*_*cpx*) of different cores. C++ pseudo code for the policy is listed in Table 3.

**Table 3:** Pseudo code of ACEP in C++.

1	compute cpx[i] for all features
2	sort descending by cpx[i]
3	initialize sum_cpx[j] = 0 (j = 1 to Ncore)
4	for feature[k] in all features
5	int addTo = 0
6	for core = 1 to Ncore
7	if sum_cpx[core] < sum_cpx[addTo]
8	addTo = core
9	add feature[k] to core[addTo]
10	sum_cpx [addTo] + = cpx[k]
11	number[addTo]++

The above pseudo-code shows the calculation procedure of algorithm complexity *cpx* for each feature. After being sorted in descending order by the attribute *cpx*, the *cpx* sums for different cores (represented by *sum*_*cpx*) are then compared, and the feature is added to the core with the lowest *sum*_*cpx*. During this process, the variable *sum*_*cpx* is updated for each feature.

## Results and Discussion

We implemented the algorithm described above in Visual Studio C++ 2010 and applied it to the center point seeking experiment for lakes in Alaska. The algorithm solves the LIC-seeking problem for an arbitrary lake polygon, especially for those that contain islands. The data include 197020 lakes, which were extracted from Landsat TM remotely sensed images. The lakes have uneven sizes and shapes, thus it is difficult for general algorithms to process such large volumes of data. We used a Dell 3.40 MHz 8-core computer with a Windows 7 64-bit operating system as our test system. The experimental results are shown in Table 4.

**Table 4:** Efficiency comparison of different data partition policies.

Time	Sequential Distribution Policy		Area Descending Policy		Algorithm Complexity Equalization Policy		ACEP except the biggest polygon	
Core No.	Features	Time(min)	Features	Time(min)	Features	Time(min)	Features	Time(min)
Core No.1	24627	**16.33**	24627	**19.43**	28150	**22.85**	24644	**19.87**
Core No.2	24628	**17.09**	24628	**20.44**	28150	**23.21**	24644	**20.12**
Core No.3	24627	**20.13**	24628	**19.65**	28150	**23.34**	24643	**20.48**
Core No.4	24628	**22.35**	24627	**22.48**	28151	**23.35**	24643	**20.49**
Core No.5	24628	**23.51**	24628	**23.88**	28150	**23.49**	24644	**20.60**
Core No.6	24627	**24.61**	24627	**25.13**	28140	**23.51**	24641	**20.61**
Core No.7	24627	**26.32**	24627	**25.32**	28128	**23.60**	24608	**20.68**
Core No.8	24628	**45.13**	24628	**39.16**	1	**30.02**	24552	**20.68**

**Fig 6:**
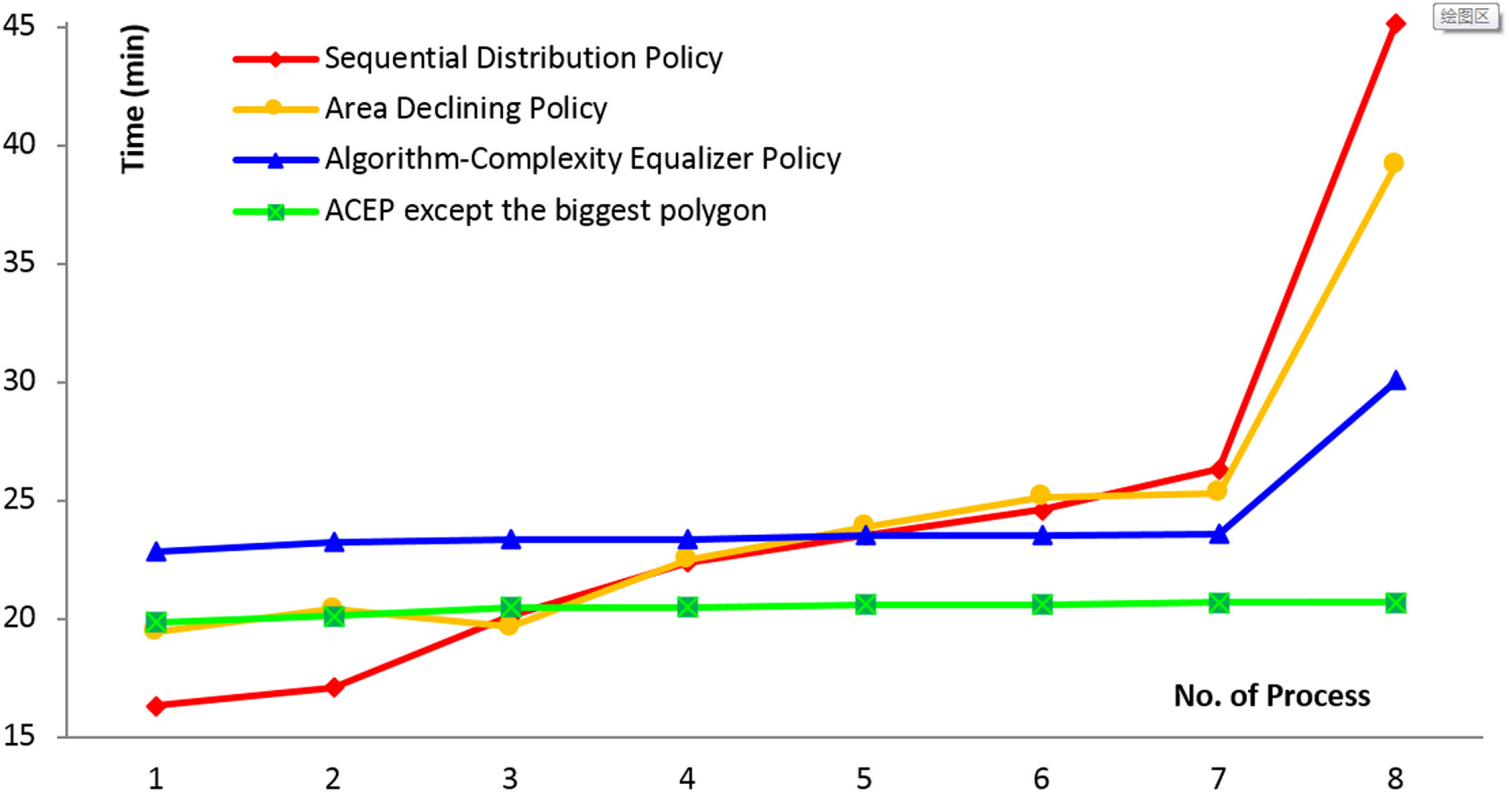
Time comparison of different data partition policy in Tab.4.

We partitioned the 197020 polygons into eight parts, according to the three methods described above. We then distributed the eight parts to eight cores and processed the features in the different cores. A comparison of the time consumption of the different policies is shown in Fig 6.

From Table 4 and Fig 6, we can see that the SDP method required 45.13 minutes, making it the slowest policy. The ADP method, which required 39.16 minutes, was also slow (similarly, the perimeter descending policy required 39.91 minutes). In contrast, ACEP only required 30.02 minutes to complete the LIC-seeking task. By examining the feature distribution numbers of the three methods from Table 4, it is evident that the SDP and ADP methods both have almost even numbers of features on each core, while for ACEP there are uneven numbers of features on each core. In fact, we aim to achieve high parallel efficiency in data processing, that is, to take full advantage of all the computer's calculation resources; therefore, the computation demands for the different cores should be as even as possible. Moreover, the processing time of core No. 8 is still longer than the others, because this core was only assigned one feature, the most complex lake polygon in this region. This polygon contains 34834 edges and 154 internal islands, its perimeter is 1187.9 km, and its area is 2618.23 km^2^. Another experiment was conducted in this region without the largest polygon (the remaining 197019 polygons were included), and the results are shown in the last two columns of Table 4. From this experiment, we can see that the numbers of features assigned to each core have changed, and the data processing times among the different cores are more even. This meets the requirement to equalize the task on different computation cores and proves the feasibility of the ACEP.

## Conclusions

This paper presented an efficient center point-seeking algorithm for LICs, to facilitate regional lake registration. First, it introduced the "divide-and-conquer" Voronoi generation method and the LIC-seeking algorithms. The following algorithm improvements were then proposed: first, the medial axis generation algorithm was presented based on the Voronoi generation method, and its simplification method was also provided to reduce LIC-seeking computations. Second, a parallel computing data processing method was proposed, based on the independency among different polygons during LIC searches, and the vector data partition policy was discussed in detail. After analyzing the efficiency of different policies, we concluded that the algorithm complexity equalization policy (ACEP) was the optimal method; this method can also be applied to other similar vector data partition policies for parallel computing.

We have determined that the algorithm only requires approximately 30 minutes to complete the deepest point estimations of 197020 Alaskan lakes, which can meet the needs of multi-phase image and lake registration. Further work will apply this algorithm to the deepest point estimation of all the extracted lakes from Landsat in the United States. There will be about five million lakes to be processed, which will further test the accuracy and efficiency of the algorithm presented in this paper.

## Abbreviations

LIC: Largest Inner Circle
MAS: Medical axis simplification
SDP: Sequential distribution policy
ADP: Attribute descending policy
ACEP: Algorithm complexity equalization policy

## References

[1] Shah CA, Sheng YW, Smith LC. Automated Image Registration Based on Pseudoinvariant Metrics of Dynamic Land-Surface Features. IEEE Transactions on Geoscience and Remote Sensing, 2008,46: 3908–3916.

[2] Joseph O’ Rourke. Computational Geometry in C (Second Edition). Cambridge University Press 2005

[3] Vincent WF, Laybourn-Parry J. Polar Lakes and Rivers, Limnology of Arctic and Antarctic Aquatic Ecosystems, Oxford university press 2008.

[4] Wang J, Sheng Y, Hinkel KM, Lyons EA. Drained thaw lake basin recovery on the western Arctic Coastal Plain of Alaska using high-resolution digital elevation models and remote sensing imagery. Remote Sensing of Environment, 119 (2012), 325–336.

[5] Lee DT. Medial Axis Transformation of a Planar Shape. IEEE Transactions on Pattern Analysis and Mechine Intelligence. 1982 4(4): 363–369 10.1109/tpami.1982.4767267 21869050

[6] Preparata P. The medial axis of a simple polygon. Proc. 6th Symp. Math. Foundations of Comput. Sci., Sept. 1977, pp.443–450.

[7] Lee DT, Drysdale RL. Generalization of Voronoi diagrams in the plane. SIAM J. Comput. 1981 10:73:87

[8] Ramamurthy R, Farouki RT. Voronoi diagram and medial axis algorithm for planar domains with curved boundaries I. Theoretical foundations. Journal of Computational and Applied Mathematics. 1999 102: 119–141

[9] Shen DY, Sheng YW. Area Pa rtitioning for Channel Network Extraction Using Digital Elevation Models and Remote Sensing. IEEE Geoscience And Remote Sensing Letters. 2012 9(2): 194–198

[10] Drysdale RL, Lee DT. Generalized Voronoi Diagrams in the plane. Proc. 16th Allerton Conf. Commun. Control Comput. 1978: 833–842

[11] Blum H. A Transformation for extracting new descriptors of shape Proc. Symp. Models for Perception of Speech and Visual Form. Whaten-Dunn W., Ed. Cambridge, MA: M.I.T. Press 1967: 362–380

[12] Leymarie FF, Benjamin KB. The medial scaffold of 3d unorganized point clouds. IEEE Trans Pattern Anal Mach Intell 2007,29(2):313–30. 17170483 10.1109/TPAMI.2007.44

[13] Borgefors G, Ragnemalm I, di Baja S. The Euclidean distance transform: finding the local maxima and reconstructing the shape, in: Procs. of the 7th Scand. Conf. on image analysis, vol. 2, 1991, pp. 974–981.

[14] Hesselink W, Roerdink J. Euclidean skeletons of digital image and volume data in linear time by the integer medial axis transform, IEEE Trans. PAMI 30 (12) (2008) 2204–2217. 10.1109/TPAMI.2008.21 18988952

[15] Hulin J. Axe médian discret: Propriétés arithmétiques et algorithmes, Ph.D. Thesis, Université Aix-Marseille II, Marseille, 2009.

[16] Chaussard J, Couprie M, Talbot H. Robust skeletonization using the discrete lambda-medial axis, Pattern Recogn. Lett. 32 (9) (2011) 1384–1394.

[17] Davies ER, Plummer APN. Thinning algorithms: a critique and a new methodology, Pattern Recogn. 14 (1–6) (1981) 53–63.

[18] Talbot H, Vincent L. Euclidean skeletons and conditional bisectors, Proceedings of VCIP’92, vol. 1818, SPIE, 1992, pp. 862–876.

[19] Serra J. Image Analysis and Mathematical Morphology, Academic Press, 1982.

[20] Attali D, Lachaud J. Delaunay conforming iso-surface, skeleton extraction and noise removal, Comput. Geom.: Theory Appl. 19 (2001) 175–189.

[21] Ogniewicz R, Kübler O. Hierarchic voronoi skeletons, Pattern Recogn. 28 (33) (1995) 343–359.

[22] Siddiqi K, Bouix S, Tannenbaum A, Zucker S. The Hamilton–Jacobi skeleton, in: International Conference on Computer Vision (ICCV), 1999, pp. 828–834.

[23] Kimmel R, Shaked D, Kiryati N, Bruckstein AM. Skeletonization via distance maps and level sets, Comput. Vis. Image Underst. 62 (1995) 382–391.

[24] Zhu H, Liu Y, Zhao J, Wang H. Calculating the medial axis of a CAD model by multi-CPU based parallel computation. Advances in Engineering Software, 85 (2015) 96–107.

[25] Oishi Y, Sugihara K. Topology Oriented Divide-and-Conquer Algorithm for Voronoi Diagrams. Graphical Models and Image Processing. 1995 57(4): 303–314

[26] Preparata P. The medial axis of a simple polygon. Proc. 6th Symposium Mathematical Foundations of Computer Science, Sept. 1977, 443–450.

[27] Drysdale RL, Lee DT. Generalized Voronoi Diagrams in the plane. 16th Annual Allerton Conference on Communication, Control and Computing, 1978, 833–842

[28] Cheonga O, Everettb H, Glisse M. Farthest-polygon Voronoi diagrams. Computational Geometry, 2011, 44: 234–247

[29] Kirkpatrick DG. Efficient computation of continuous skeletons. Proc. 20th Annu. Symp. Found. Computer Sci, 1979, 18–27

[30] Dey TK, Zhao W. Approximate medial axis as a Voronoi subcomplex. Computer-aided Design. 2004 36: 195–202

[31] Beristain AM, Gonzalez AI. A Pruning Algorithm for Stable Voronoi Skeletons. Journal of Mathematical Imaging and Vision. 2012 42:225–237

[32] Dorado R. Medial axis of a planar region by offset self-intersections. Computer-aided Design. 2009 41: 1050–1059

[33] Shen ZF, Luo JC, Chen QX, Huang GY. Sheng H. Data partition policy of high resolution remotely sensed image parallel processing. Journal of Harbin Institute of Technology. 2006 38(11): 1968–1973

[34] Shen ZF, Luo JC, Wu W, Hu XD. A New Approach to Improve the Cluster-based Parallel Processing Efficiency of High-Resolution Remotely Sensed Image. Journal of the Indian Society of Remote Sensing. 2012 40(3):357–370

